# Non-digestible oligosaccharides scFOS/lcFOS facilitate safe subcutaneous immunotherapy for peanut allergy

**DOI:** 10.1186/s12948-019-0111-5

**Published:** 2019-04-04

**Authors:** Laura Wagenaar, Manon van Roest, Laura J. W. Kruijssen, Peter J. Simons, Louis Boon, Marlotte M. Vonk, Betty C. A. M. van Esch, Leon M. J. Knippels, Johan Garssen, Raymond H. H. Pieters, Joost J. Smit

**Affiliations:** 1 Department of Immunotoxicology, Faculty of Veterinary Medicine, Institute for Risk Assessment Sciences, Utrecht University, Yalelaan 104, 3508 TD Utrecht, The Netherlands; 2 Department of Pharmacology, Faculty of Science, Utrecht Institute for Pharmaceutical Sciences, Utrecht University, Utrecht, The Netherlands; 3 Department of Immunology, Danone Nutricia Research, Utrecht, The Netherlands; 4 Bioceros BV, Utrecht, The Netherlands

**Keywords:** Peanut allergy, Immunotherapy, Mast cells, Non-digestible oligosaccharides

## Abstract

**Background:**

Improving the safety of subcutaneous immunotherapy (SCIT) for food allergy is necessary to reduce side effects and achieve long-term tolerance. We determined the effect of dietary supplementation with 1% non-digestible short- and long-chain fructo-oligosaccharides (scFOS/lcFOS) on safety and efficacy of SCIT using a peanut allergy mouse model.

**Methods:**

After sensitization, mice received a scFOS/lcFOS or control diet for the rest of the study. To study safety of SCIT, mice were dosed with a single subcutaneous injection of peanut extract (PE) or PBS. To study efficacy, mice were dosed subcutaneously (SCIT, 3 times/week) with PE or PBS for 3 weeks. Hereafter, acute allergic skin responses, anaphylactic shock symptoms and body temperature were assessed. To study the mechanism in vitro, the human IgE receptor (FcεRI)-transfected rat mast cell (RBL) line was sensitized with an oligoclonal pool of chimeric human (chu)IgE antibodies against bovine β-lactoglobulin (BLG) and incubated with the oligosaccharides before exposure to BLG to assess direct the effect on degranulation.

**Results:**

scFOS/lcFOS reduced anaphylaxis caused by a single PE SCIT dose. scFOS/lcFOS alone also reduced the acute allergic skin response. Moreover, scFOS/lcFOS supplementation resulted in lower MMCP-1 levels in serum after PE SCIT dose compared to control diet, while antibody levels were not affected by the diet. In vitro incubation with scFOS/lcFOS at 0.5% suppressed the degranulation of IgE-sensitized RBL cells. However, dietary supplementation with scFOS/lcFOS did not improve the efficacy of SCIT.

**Conclusions:**

We show that scFOS/lcFOS diet improves the safety of SCIT, as evidenced by lower anaphylactic responses without compromising the efficacy in a mouse model for peanut allergy. This effect is likely to result from the suppression of mast cell effector function.

## Background

Food allergy is a major public health issue in Western countries, as it affects 8% of American children, of which most of them are peanut allergic (25%) [1]. Currently, food allergy can only be managed by strict avoidance of the causative food and in case of accidental exposure, with anaphylactic rescue medication. Therefore, a safe therapy leading to persistent tolerogenic protection is highly needed.

For many years, desensitization and/or tolerance induction to allergens via allergen-specific immunotherapy (AIT) has been the focus of research. AIT using the subcutaneous, oral, or sublingual route provided encouraging results in food allergy, despite serious and significant safety concerns [2–8]. Two small studies in peanut allergic patients showed that subcutaneous immunotherapy (SCIT) was associated with reduced symptoms [9, 10]. However, a high rate of serious systemic reactions (13%) made this treatment unsafe for routine use. Because of these limitations, although effective, it is currently not recommended to use immunotherapy for peanut allergy for routine clinical use [11–13].

Combining AIT with a nutritional intervention may provide a new window of opportunity to improve the efficacy and safety of AIT for food allergic patients. The combination of SCIT or sublingual immunotherapy for allergic rhinitis or asthma with bacterial products or Toll-like receptor ligands has demonstrated enhanced and persistent beneficial effects in both animals [14–16] and patients [17, 18]. However, human data concerning the additive effect of supplementation with immunomodulatory food components on the safety and efficacy of OIT for food allergy is limited. In peanut allergic patients, combining OIT with a probiotic strain resulted in a long-lasting clinical benefit [19, 20]. Besides probiotics, prebiotic components like dietary non-digestible oligosaccharides, derived from vegetable or dairy sources, also support growth of beneficial bacteria in the gut [21]. These oligosaccharides, like short- and long-chain fructo-oligosaccharides (scFOS/lcFOS), were able to effectively prevent the onset of allergy, and prevent allergic manifestations in different mouse models [22–24]. Moreover, in whey-sensitized mice a diet supplemented with scFOS/lcFOS showed improved efficacy of OIT [25]. Therefore, non-digestible oligosaccharides administered after sensitization but before SCIT, might provide a better safety profile during treatment and therefore might improve the efficacy of therapy.

A food allergic reaction is induced by the fast, local and systemic release of inflammatory mediators such as histamine, serotonin, and various pro-inflammatory cytokines from mast cells and basophils [26]. Consequently, we hypothesized that scFOS/lcFOS could have an effect on the basophil effector function.

In the current study, it was investigated whether dietary supplementation with scFOS/lcFOS can maintain the effectiveness in the absence of side-effects of SCIT by reducing the allergic response seen after a single SCIT dose was administered. Moreover, it was discovered that these oligosaccharides have a direct inhibitory effect on degranulation of mast cells.

## Materials and methods

### Mice

Female C3H/HeOuJ mice (5–6-week-old) purchased from Charles River Laboratories (Erkrath, Germany) were maintained under controlled conditions (relative humidity of 50–55%, 12 h light/dark cycle, temperature of 23 ± 2 °C). The mice were housed at the animal facility of Utrecht University in filter-topped macrolon cages (n = 6–8 per cage/group), with wood chipped bedding, tissues and a plastic shelter and food and water were provided ad libitum. An independent ethics committee for animal experimentation (the Ethical Committee of Animal Research of Utrecht University, Utrecht, The Netherlands) approved animal procedures. All procedures complied with the principles of good laboratory animal care following the European Directive for the protection of animals used for scientific purposes.

### Reagents and diets

Peanut protein extract (PE, 30 mg/ml) was prepared from raw peanuts (provided by Intersnack Nederland BV, The Netherlands) as described previously [27], checked for protein content by BCA analysis (Pierce, IL) and kept at − 20 °C until use. The adjuvant cholera toxin (CT) was acquired from List Biological Laboratories Inc. (Campbell, CA, USA).

Ssniff Spezialdiäten (Soest, Germany) composed the semi-purified peanut protein-free AIN-93G-based diets. The scFOS/lcFOS diet was supplemented with non-digestible oligosaccharides, which consisted of a 9:1, 1% (w/w) mixture of short-chain fructo-oligosaccharides (scFOS: oligofructose; Raftilose P95, Orafti, Wijchen, the Netherlands; > 95% degree of polymerization [DP] < 6) and long-chain fructo-oligosaccharides (lcFOS: long chain inulin; Raftiline HP, Orafti, Wijchen, the Netherlands; average DP 23 or higher, < 1% DP < 5) derived from chicory inulin. The AIN-93G diet without scFOS/lcFOS supplementation was used as control diet. The diets were stored at 4 °C prior to use.

### Experimental designs

**Fig. 1:**
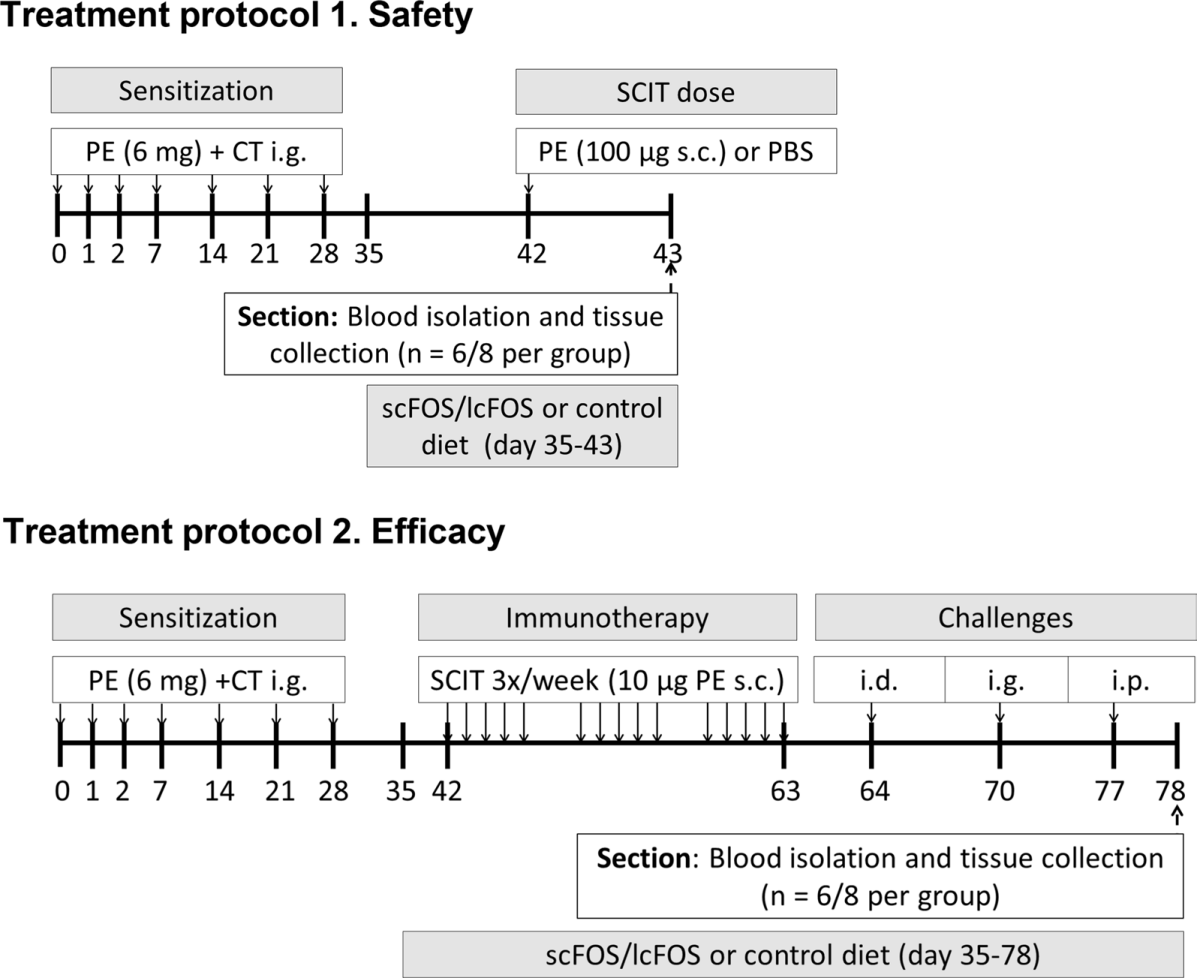
Schematic overviews of the experimental set-ups. Treatment protocol 1 Safety To study the ability of scFOS/lcFOS to improve the safety of SCIT in PE sensitized mice. Treatment protocol 2 Efficacy To study the ability of scFOS/lcFOS to improve the efficacy of SCIT in PE sensitized mice. PE, peanut extract; CT, cholera toxin; SCIT, subcutaneous immunotherapy; s.c., subcutaneous; i.d., intradermal; i.g., intragastric; i.p., intraperitoneal

Upon arrival, mice were randomly divided over the control and experimental groups and were fed control diet. To study the potential of scFOS/lcFOS to improve the safety and efficacy of SCIT, two study treatment protocols were used (Fig. 1). In both protocols, mice (n = 6–8), were sensitized to PE i.g. (6 mg PE, 200 μl/mouse) with CT (15 μg/mouse) on three consecutive days, followed by a weekly dosing for 4 weeks (PE), as previously described [27]. Sham-sensitized mice received CT in PBS alone.

The effect of scFOS/lcFOS on the safety of SCIT was studied in a peanut allergy mouse model (treatment protocol 1, Fig. 1). Depicted groups received the scFOS/lcFOS supplemented diet from day 35 until the end of the study. On day 42, anaphylactic shock symptom scores, body temperature levels and mucosal mast cell degranulation after one high dose of SCIT (PE 100 µg s.c. or PBS) were determined. At the end of the study (day 43), mice were killed by cervical dislocation and spleen was collected.

The effect of scFOS/lcFOS on the efficacy of SCIT was studied in the same peanut allergy mouse model (treatment protocol 2, Fig. 1). After sensitization, mice were treated for 3 weeks with PE, s.c. (10 µg PE/mouse) three times a week. Sham-sensitized and PE-sensitized control mice were treated i.g. with PBS alone. The diet of selected groups was supplemented with scFOS/lcFOS from day 28 (after sensitization) until the end of the study. On day 64, the acute allergic skin response was determined in all mice after i.d. exposure to PE in both ear pinnae with 1 µg PE in 20 μl PBS. On day 70, MMCP-1 levels were determined in blood samples collected 30 min after mice were i.g. exposed to 15 mg PE in 500 µl PBS. To measure ear thickness, in duplicate prior to and 1 h after i.d. PE exposure in both ear pinnae, all mice were anesthetized using inhalation of isoflurane. To determine Δ ear swelling as a measure for the acute allergic skin response, basal ear thickness (μm) was subtracted from the ear thickness 1 h post-challenge. Anaphylactic shock symptom scores and body temperature levels were determined after i.p. exposure on day 77 with 100 µg PE in 200 µl PBS. Body temperature was measured every 10 min after the i.p. or high SCIT and OIT challenge using a rectal thermometer and clinical symptoms were scored after 40 min, according to the method described by Li et al. [28]. At the end of the experiment on day 78, the mice were killed by cervical dislocation and blood and organs were collected.

### Serum levels of MMCP-1 and allergen-specific IgE, IgA, IgG1 and IgG2a

PE-specific IgA, IgE, IgG1 and IgG2a levels in serum were detected by ELISA as previously described [27]. Briefly, high-binding 96-wells plates (Costar 3590, Corning Incorporated, Corning, NY, USA) were coated at 4 °C with 10 µg/ml PE in PBS (IgG1 and IgG2a) or with 1 µg/ml rat anti-mouse IgE or IgA (BD Biosciences, Alphen aan den Rijn, The Netherlands) in PBS ON. Plates were blocked with 0.5% BSA-ELISA buffer for 1 h (RT). Diluted serum samples were incubated for 2 h (RT). For detection, AP-coupled anti-IgG1 and IgG2a antibodies were incubated for 1 h (RT). Subsequently, 1 mg/ml p-nitrofenylphosphat in diethanolamine buffer was used for the color reaction, which was stopped with a 10% EDTA solution. To measure PE-specific IgE and IgA, PE-DIG conjugate solution (1 h RT) and peroxidase-conjugated anti-DIG fragments (1 h at RT in the dark) were added. After incubation, a tetramethylbenzidine substrate solution was used and the color reaction was stopped with 2 M H_2_SO_4_. Absorbance was measured at 405 nm (IgG1 and IgG2a) and at 450 nm (IgE and IgA) using an Asys expert 96 plate reader (Biochrom, Cambourne, UK). Concentrations of IgE, IgA, IgG1 and IgG2a were calculated in arbitrary units (AU) using a standard curve of pooled sera from PE-sensitized mice.

Serum obtained 30 min after i.g. challenge (treatment protocol 1 and 2, Fig. 1) was used to measure Mouse Mast Cell Protease-1 (MMCP-1). MMCP-1 was determined by using an MMCP-1 Sandwich ELISA kit (eBioscience MMCP-1 ELISA Ready-SET-Go Kit, Breda, The Netherlands) according to the manufacturer’s instructions.

### Cytokine release after ex vivo stimulation of spleen lymphocytes with PE

8 × 10^5^ cells derived from spleen were cultured (200 μl/well) in U-bottom culture plates (Greiner, Frickenhausen, Germany) using RPMI 1640 medium (Lonza, Verviers, Belgium) with 10% FCS, penicillin (100 U/ml)/streptomycin (100 μg/ml) (Sigma). All cells were stimulated with culture medium as a negative control, a polyclonal stimulation with anti-CD3/CD28 (1 μg/ml, clone 145-2C11 and clone 37.51, eBioscience) or allergen-specific stimulation with PE (100 μg/ml). Interleukin (IL)-5, IL-10, IL-13 and Interferon-γ (IFN-γ) production by T cells were determined after 48 h (anti-CD3/CD28) or 96 h (PE) incubation. Culture supernatants were collected and stored at − 20 °C until further analysis with the Ready-SET-Go!^®^ ELISA (eBioscience) according to the manufacturer’s instructions.

### RBL cell degranulation assay

Human FcεRI-expressing rat basophilic leukemia RBL-SX38 cells transfected with a nuclear factor of activated T-cells (NFAT)-responsive luciferase reporter gene, were used to measure mast cell degranulation as previously described [29]. In short, these RBL cells were plated in clear bottom 96 well plates and sensitized using an oligoclonal pool of chimeric human (chu)IgE antibodies against bovine β-lactoglobulin (BLG, a major allergen in bovine whey), as described by Knipping et al. [30]. Hereafter, RBL cells were incubated for 24 h with 0.05 and 0.5% scFOS/lcFOS (ratio 9:1). To induce degranulation, cells were exposed to 1, 10, 100 and 1000 ng/ml BLG. After stimulation, luciferase substrate solution containing cell lysis reagent (One-Glo, Promega Corp., Tokyo, Japan) was added to the cells, and chemiluminescence was measured. Luciferase expression levels are represented as the fold increase of relative light units compared with the background expression, after subtraction of a blank control (without cells).

### Statistics

For all statistical analyses, GraphPad Prism 6.0c software for Macintosh (GraphPad Software, San Diego, CA, USA) was used. Anaphylaxis symptom scores and cytokine levels were analyzed using Kruskal–Wallis test for nonparametric data with Dunn’s post hoc test. Body temperature levels of the peanut allergy safety study were analyzed on each time-point by one-way ANOVA and Bonferroni’s post hoc test to compare preselected groups. Body temperature levels for the efficacy study were analyzed using a one-way repeated measures ANOVA and Bonferroni’s post hoc test. The acute allergic skin response was statistically analyzed by one-way ANOVA and Bonferroni’s post hoc test for multiple comparisons to compare preselected groups. Serum MMCP-1 results were log-transformed and statistically analyzed by one-way ANOVA and Bonferroni’s post hoc test for multiple comparisons to compare preselected groups. Immunoglobulin levels were depicted as mean ± SEM, were log transformed prior to testing, and statistical difference compared to the PE-sensitized control treatment was analyzed each day by a one-way ANOVA and Dunnett’s post hoc test. All data are presented as mean ± SEM of 5–8 mice per group and results were considered statistically significant when P < 0.05.

## Results

### scFOS/lcFOS reduced anaphylaxis and mast cell degranulation after SCIT PE dose

**Fig. 2:**
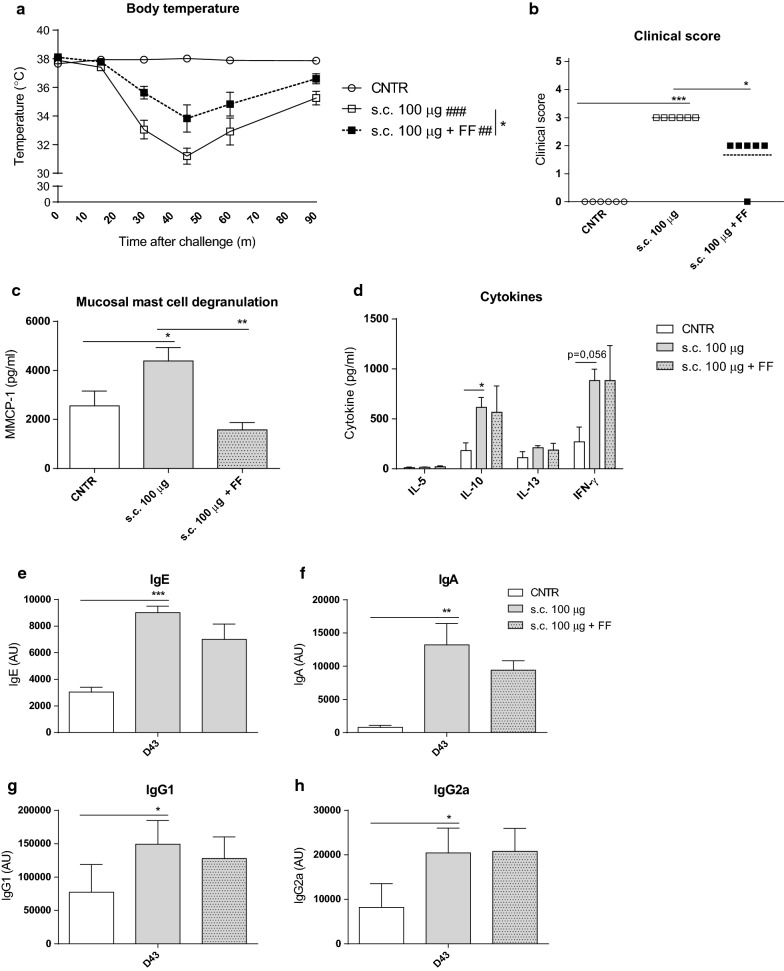
Allergic manifestations evaluated in PE-sensitized mice after fed the scFOS/lcFOS supplemented diet and a singular SCIT dose. The effect of scFOS/lcFOS on the safety of SCIT in a peanut allergy model, according to treatment protocol 1 (Fig. 1). **a** Change in body temperature after SCIT challenge on day 42. **b** Anaphylactic shock symptom scores determined 40 min after SCIT challenge on day 42. **c** Concentrations of MMCP-1 in serum collected 30 min after SCIT challenge on day 42. **d** Cytokine concentrations (IL-5, IL-10, IL-13 and IFN-γ) after ex vivo stimulation of splenocytes with PE collected at day 43. **e**–**h** Allergen-specific IgE, IgA, IgG1 and IgG2a measured by ELISA in serum of mice. Data are represented as mean ± SEM n = 6 mice/group, experiments were performed twice independently. Statistical analysis was performed using a two-way repeated measures ANOVA with Bonferroni’s post hoc test (body temperature), a one-way ANOVA with Bonferroni’s post hoc test (MMCP-1 and antibody levels), or a Kruskal–Wallis test with Dunn’s post hoc test (Clinical score and cytokine levels). For body temperature; ^####^P < 0.0001; ^##^P < 0.01 compared to the control group. **P < 0.01 compared to same group on control diet. For other results; ***P < 0.001; **P < 0.01; *P < 0.05 compared to indicated group. s.c., subcutaneous; i.g. intra gastric; PE, peanut extract; CNTR, control group; FF, scFOS/lcFOS; MMCP-1, mucosal mast cell protease-1; IL-, interleukin; IT, immunotherapy

The protective effect of scFOS/lcFOS supplementation to reduce side effects induced by SCIT was examined by analyzing allergic responses after one SCIT PE dose (Fig. 2). A single dose of 100 µg s.c. caused an anaphylactic response in PE-sensitized mice, measured by a severe drop in body temperature and high anaphylactic symptom scores compared to the mice receiving a PBS dose (Fig. 2a, b). Importantly, mice that were supplemented with scFOS/lcFOS after sensitization had a significant lower anaphylactic response (Fig. 2a, b).

In addition, exposure of PE-sensitized mice to a 100 µg SCIT dose after supplementation with scFOS/lcFOS resulted in lower serum MMCP-1 levels compared to the group fed the control diet (Fig. 2c).

Cytokine production was measured in culture supernatant of PE-restimulated splenocytes obtained on day 43 (Fig. 2d). Splenocytes from SCIT-treated mice, both in the presence and absence of scFOS/lcFOS, showed an increased PE-induced IL-10 production when compared to control-treated mice (Fig. 2d). scFOS/lcFOS feeding did not change the cytokine production (Fig. 2d). In addition, scFOS/lcFOS feeding of PE-sensitized mice did not change antibody levels in serum compared to mice on control diet (Fig. 2e, f).

Noticeably, scFOS/lcFOS supplementation after sensitization considerably reduced side effects of SCIT possibly by an inhibitory effect on mast cell degranulation.

### Incubation with 0.5% scFOS/lcFOS lowered RBL degranulation

**Fig. 3:**
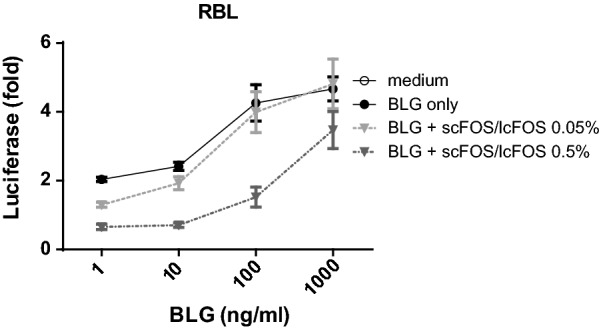
RS-ALT8 cell degranulation. RS-ATL8 cells were sensitized with oligoclonal pool of chimeric human (chu)IgE antibodies and incubated with scFOS/lcFOS. IgE crosslinking-induced luciferase expression by 1, 10, 100 and 1000 mg/ml BLG or medium was shown. Data are represented as mean ± SEM (n = 2 for 3 separate experiments)

Since there was indication based on MMCP-1 levels that mast cells were involved in this reduction, we next focused on the effect of these oligosaccharides on degranulation of basophils in vitro. scFOS/lcFOS dose-dependently inhibited the BLG-induced degranulation of anti-BLG IgE-sensitized RBL cells (Fig. 3).

### SCIT was able to effectively induce protection against anaphylaxis without improving effect of the dietary intervention

**Fig. 4:**
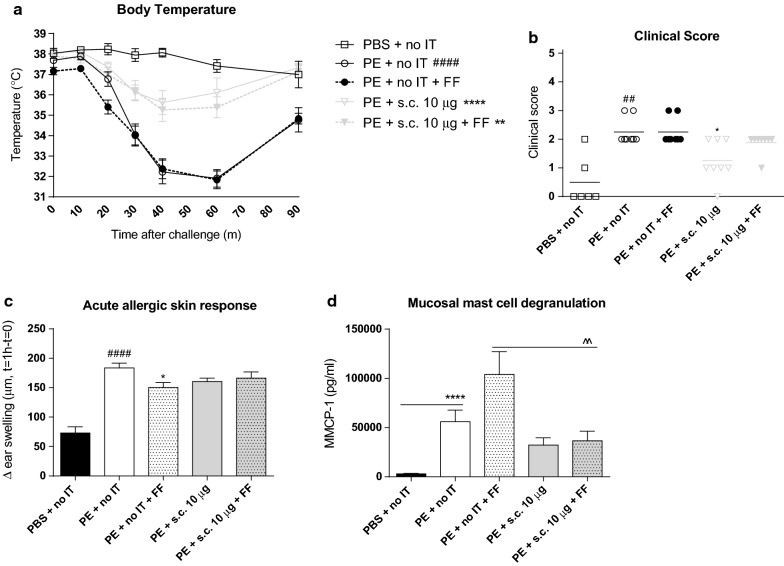
Allergic manifestations evaluated in PE-sensitized mice after having received SCIT or SCIT in combination with scFOS/lcFOS. The effect of scFOS/lcFOS on the efficacy of SCIT, according to treatment protocol 2 (Fig. 1). **a** Change in body temperature after intraperitoneal challenge on day 77. **b** Anaphylactic shock symptom scores determined 40 min after intraperitoneal challenge on day 77. **c** Acute allergic skin response measured as Δ ear swelling 1 h after intradermal challenge on day 64. Δ Ear swelling was calculated as average of left and right ear swelling subtracted with the basal ear thickness before challenge. **d** Concentrations of MMCP-1 in serum collected 30 min after intragastric challenge on day 70. Data are represented as mean ± SEM n = 6–8 mice/group. Statistical analysis was performed using a two-way repeated measures ANOVA with Bonferroni’s post hoc test (body temperature) one-way ANOVA and Bonferroni’s post hoc test (acute allergic skin response and MMCP-1) or a Kruskal–Wallis test with Dunn’s post hoc test (Clinical score). ^####^P < 0.0001; ^##^P < 0.01 compared to sham-sensitized control. ****P < 0.0001; **P < 0.01; *P < 0.05 compared to PE-sensitized control. s.c., subcutaneous; PE, peanut extract; FF, scFOS/lcFOS; MMCP-1, mucosal mast cell protease-1; IT, immunotherapy

The effect of scFOS/lcFOS on the efficacy of SCIT was examined by analyzing allergic responses after various PE exposures (Fig. 4). In PE-sensitized control mice, the i.p. challenge with PE elicited an anaphylactic response compared to the sham-sensitized control mice (Fig. 4a, b). This anaphylactic response was characterized by a sharp drop in body temperature and high clinical symptom scores. SCIT, with or without scFOS/lcFOS, resulted in a lower anaphylactic drop in body temperature compared to the PE-sensitized control mice, whereas scFOS/lcFOS alone did not (Fig. 4a). Moreover, SCIT effectively decreased anaphylactic symptom scores compared to the non-treated PE-sensitized mice (Fig. 4b).

The i.d. PE-challenge resulted in an increased acute allergic skin response, as characterized by an ear swelling response 1 h after injection, in PE-sensitized control mice compared to sham-sensitized control mice (Fig. 4c). SCIT treatment did not change ear swelling compared to the PE-sensitized control mice (Fig. 4c). However, scFOS/lcFOS dietary supplementation resulted in a reduced ear swelling compared to the PE-sensitized control group again showing reduction of the allergic response (Fig. 4c).

Levels of MMCP-1, reflecting mucosal mast cell responses, were measured in serum collected 30 min after i.g. challenge (day 70, Fig. 4d). MMCP-1 levels were increased in PE-sensitized control mice compared to PBS-sensitized control mice (Fig. 4d). SCIT treatment in combination with scFOS/lcFOS resulted in lower MMCP-1 levels after i.g. challenge compared to only scFOS/lcFOS dietary intervention (Fig. 4d).

### SCIT increased IgE and IgG levels

**Fig. 5:**
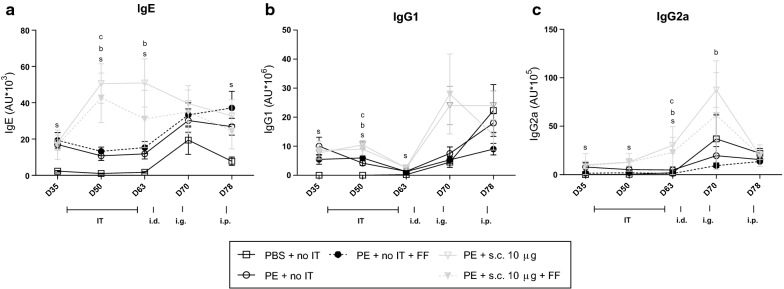
PE specific antibody levels in serum. **a**–**c** Allergen-specific IgE, IgG1 and IgG2a measured by ELISA in serum of mice from treatment protocol 2 (Fig. 1). Data are represented as mean ± SEM n = 6–8 mice/group. Statistical analysis was performed, after log transformation, using a one-way ANOVA and Bonferroni’s post hoc test (on each day). All treatment groups were compared to the PE-sensitized control group and significant differences were indicated with letters: a P < 0.05; aa P < 0.01; aaa P < 0.001; aaaa P < 0.0001. Letters used: s for PBS-sensitized control group; a for scFOS/lcFOS control group; b for SCIT; c for SCIT plus scFOS/lcFOS. s.c., subcutaneous; i.g. intra gastric; i.p., intraperitoneal challenge; PE, peanut extract; FF, scFOS/lcFOS; IT, immunotherapy

On different time-points antibody concentrations were determined (Fig. 5). PE-sensitized mice showed enhanced IgE, IgG1 and IgG2a levels compared to the sham-sensitized mice (day 35, 50, 63, Fig. 5a–c). SCIT, with or without scFOS/lcFOS, first increased IgE and IgG1 and later IgG2a levels compared to non-treated PE-sensitized mice (day 50 and 63, Fig. 5a–c). The i.g. challenge induced IgG2a levels in SCIT-treated mice compared to non-treated PE-sensitized mice (day 70, Fig. 5c). After the i.p. challenge IgE levels increased in non-treated PE-sensitized mice but SCIT treatment protected against this increase (day 78, Fig. 5a).

### SCIT induced Th2 cytokine production in spleen

**Fig. 6:**
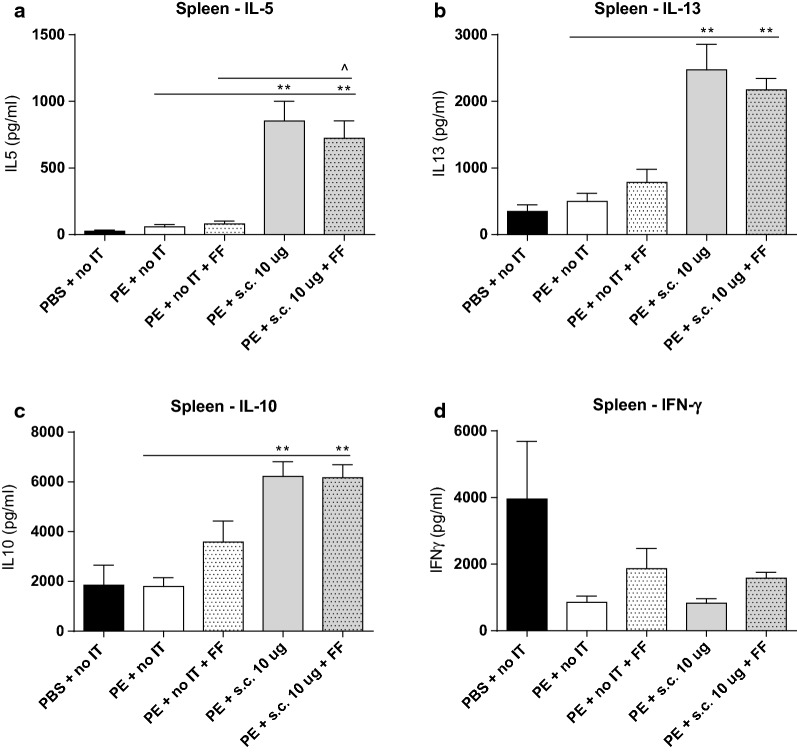
Cytokine concentrations after ex vivo stimulation of spleen-derived lymphocytes with PE, determined by ELISA. Spleen-derived lymphocytes from day 78 were cultured for 96 h in the presence of PE or medium (treatment protocol 2, Fig. 1, medium data not shown). **a** IL-5 concentration. **b** IL-13 concentration. **c** IL-10 concentration. **d** IFN-γ concentration. Data are represented as mean ± SEM n = 6–8 mice/group. Statistical analysis was performed using Kruskal–Wallis test with Dunn’s post hoc test. **P < 0.01 compared to PE-sensitized control. ^^^P < 0.05 compared to scFOS/lcFOS control. IT, immunotherapy; s.c., subcutaneous; PE, peanut extract; IL-, interleukin; FF, scFOS/lcFOS

To further study the effects of the treatments in reducing allergic symptoms, cytokine production by T cells was studied. No differences were found between cytokine concentrations upon allergen-specific stimulation of lymphocytes derived from PE-sensitized control mice and sham-sensitized control mice (Fig. 6a–d). scFOS/lcFOS supplementation did not affect the cytokine production (Fig. 6a–d). SCIT treatment, with or without the scFOS/lcFOS, increased IL-5, IL-10 and IL-13 production by PE-stimulated splenic lymphocytes when compared to PE-sensitized control mice (Fig. 6a–c), showing that scFOS/lcFOS did not impact the immunological modulation induced by SCIT.

## Discussion

To reduce side-effects while maintaining efficacy of AIT for food allergy, several new treatment concepts have been investigated, as reviewed in [31–34]. The combination of AIT with nutritional interventions may provide a new possibility to enhance the safety and efficacy of AIT due to a reduction in side-effects during dose increasing protocols. Our results indicate that supplementation of the diet with scFOS/lcFOS reduced the side effects caused by a single PE SCIT dose. This effect appears to be due to a direct effect on mast cells, since scFOS/lcFOS reduced mucosal mast cell degranulation in vivo and inhibited degranulation of RBL cells in vitro. These results support the concept that nutritional interventions have the potential to improve safety of SCIT.

The immunologic mechanisms underlying AIT are not fully understood and the observed desensitization or tolerance induction by AIT can occur via various, interrelated pathways [35]. Evidence from mouse models have shown that tolerance induction by OIT may be due to the activation of Tregs, i.e. CD4+CD25+FoxP3+ cells and IL-10- and TGF-β-producing Tregs [25, 36]. This is reflected in OIT treated peanut allergic patients by significant changes in antigen induced T-cell function and demethylation FoxP3 CpG sites after OIT [37]. In the used mouse model, OIT induced allergen-specific IgA and IgG1 antibodies [25, 38, 39]. Recently, IgG4 and IgA antibodies have been shown to be prominent immunoglobulins in the sustained regulation of food allergies in human immunotherapy trials [6, 40]. In a murine model, mast cell degranulation and IgE-mediated systemic anaphylaxis induced by allergen ingestion were suppressed by allergen-specific IgG antibodies in the serum [41]. Accordingly, in a human/mouse chimeric model of respiratory allergy, it was demonstrated that post‐AIT sera containing AIT‐induced blocking antibodies is able to ameliorate allergic airway responses [42]. This suppressive effect of IgG might explain the ability of SCIT to reduce anaphylaxis after i.p. challenge.

This efficacy of SCIT to lower allergic manifestations, as shown by the decreased anaphylaxis after i.p. challenge, was not affected by scFOS/lcFOS. Peanut allergy in humans is Th2-dependent [43], during sensitization to peanut, priming of allergen-specific Th2 cells results in the production of Th2 cytokines (such as IL-4 and IL-13), which are responsible for class switching by B cells, allowing IgE production. In the current model, SCIT treatment showed a clear induction of cytokine production of IL-5, IL-13 and IL-10 by spleen-derived lymphocytes. These data may suggest that SCIT does not depend on reducing the Th2 response, but rather on inducing the regulatory T cell response, which remained unaffected by scFOS/lcFOS.

Using a food allergy mouse model, it was shown that oral treatment with probiotics is able to reduce both systemic and local anaphylactic symptoms induced by oral challenge with the sensitizing allergen shrimp tropomyosin [44]. Similar effects have been demonstrated in clinical trials, showing that probiotics are able to modulate the mucosal immune response, and that specific strains, especially lactic acid bacteria, are able to reduce allergic symptoms [45, 46]. Besides probiotics, also prebiotic oligosaccharides are able to reduces the incidence of atopic dermatitis [47, 48]. However, studies using prebiotics for food allergy are limited, and the mechanism of clinical benefit is still unknown. Importantly, non-digestible oligosaccharides are detected in serum and urine of piglets fed galacto-oligosaccharides, meaning they can cross the gut epithelial barrier and could directly affect immune cells [49]. We show that 0.5% scFOS/lcFOS inhibit degranulation upon allergen challenge in vivo and in vitro. These findings are in line with those of Xu et al. who show that treatment with sulfated oligosaccharides, extracted from *Eucheuma (E.) cottonii*, lowered the serum levels of MMCP-1 after challenge in tropomyosin-allergic mice [50]. Moreover, these oligosaccharides were able to inhibit the secretion of allergy-related mediators like β-hexosaminidase, histamine and IL-4 and TNF-α [50].

We and others hypothesized that the combination of AIT with immunomodulatory components, which addresses both specific and unspecific (e.g. linked to innate effector cells such as mast cells) modulation of the immune response respectively, could be a promising strategy to improve efficacy and safety of AIT. Recently, we have shown that oligosaccharides are capable of improving the efficacy of OIT [39], and here we show that these substances also improve safety of SCIT. Previously it was shown that combining subcutaneous or sublingual immunotherapy with bacterial adjuvants or Toll-like receptor ligands could enhance tolerogenicity in allergic rhinitis [14–16, 51, 52], a strategy which is already successfully translated to humans [18, 53–55]. In peanut allergic children, OIT administration was combined with the probiotic *Lactobacillus rhamnosus* [19]. The authors report a long-lasting clinical benefit and persistent sustained unresponsiveness to peanut after 4 years without treatment [20]. Although no control (only OIT) was included, the proportion of children experiencing adverse events was lower compared to other trials where only OIT was used [19, 56, 57]. This study suggests that the combination of OIT and probiotics may lower the incidence of adverse events making this treatment clinically feasible.

## Conclusions

In summary, we show that scFOS/lcFOS reduced anaphylaxis caused by a single PE SCIT dose, hereby improving the safety profile of SCIT in a mouse model. However, scFOS/lcFOS was not able to further improve the efficacy of SCIT in the current protocol. Nevertheless, when side-effects are reduced higher dose of SCIT can be used for tolerance induction. Translated to clinical practice, the improvement of the safety profile could facilitate SCIT for peanut allergic patients more appropriate, although further studies are needed to determine the long-term supportive role of scFOS/lcFOS for AIT.

## Abbreviations

AIT: allergen-specific immunotherapy
PE: peanut extract
OIT: oral immunotherapy
SCIT: subcutaneous immunotherapy
i.g.: intragastric
CT: cholera toxin
i.d.: intradermal
i.p.: intraperitoneal
Ig: immunoglobulin
MMCP-1: murine mast cell protease-1
IT: immunotherapy
Foxp3: forkhead box protein 3
Th: T helper
IL: interleukin
DP: degree of polymerization
IFNγ: interferon γ
